# Influence of CeO_2_ and TiO_2_ Particles on Physicochemical Properties of Composite Nickel Coatings Electrodeposited at Ambient Temperature

**DOI:** 10.3390/ma15165550

**Published:** 2022-08-12

**Authors:** Iryna Makarava, Mohammadamin Esmaeili, Dzmitry S. Kharytonau, Leonardo Pelcastre, Jacek Ryl, Mohammad Reza Bilesan, Esa Vuorinen, Eveliina Repo

**Affiliations:** 1 Department of Separation Science, School of Engineering Science, LUT University, Yliopistonkatu 34, FI-53850 Lappeenranta, Finland; 2 Helmholtz-Zentrum Dresden-Rossendorf, Helmholtz Institute Freiberg for Resource Technology, 09599 Freiberg, Germany; 3 Soft Matter Nanostructures Group, Jerzy Haber Institute of Catalysis and Surface Chemistry, Polish Academy of Sciences, Niezapominajek 8, PL-30239 Krakow, Poland; 4 Division of Machine Elements, Luleå University of Technology, Regnbågsallén, SE-97187 Luleå, Sweden; 5 Institute of Nanotechnology and Materials Engineering, Faculty of Applied Physics and Mathematics, Gdansk University of Technology, Narutowicza St. 11/12 Gdansk, PL-80233 Gdansk, Poland; 6 Division of Materials Science, Luleå University of Technology, Regnbågsallén, SE-97187 Luleå, Sweden

**Keywords:** nickel, composite coating, TiO_2_, CeO_2_, corrosion

## Abstract

The Ni-TiO_2_ and Ni-CeO_2_ composite coatings with varying hydrophilic/hydrophobic characteristics were fabricated by the electrodeposition method from a tartrate electrolyte at ambient temperature. To meet the requirements of tight regulation by the European Chemicals Agency classifying H_3_BO_3_ as a substance of very high concern, Rochelle salt was utilized as a buffer solution instead. The novelty of this study was to implement a simple one-step galvanostatic electrodeposition from the low-temperature electrolyte based on a greener buffer compared to traditionally used, aiming to obtain new types of soft-matrix Ni, Ni-CeO_2_, and Ni-TiO_2_ coatings onto steel or copper substrates. The surface characteristics of electrodeposited nickel composites were evaluated by SEM, EDS, surface contact angle measurements, and XPS. Physiochemical properties of pure Ni, Ni-CeO_2,_ and Ni-TiO_2_ composites, namely, wear resistance, microhardness, microroughness, and photocatalytic activity, were studied. Potentiodynamic polarization, EIS, and ICP-MS analyses were employed to study the long-term corrosion behavior of coatings in a 0.5 M NaCl solution. Superior photocatalytic degradation of methylene blue, 96.2% after 6 h of illumination, was achieved in the case of Ni-TiO_2_ composite, while no substantial change in the photocatalytic behavior of the Ni-CeO_2_ compared to pure Ni was observed. Both composites demonstrated higher hardness and wear resistance than pure Ni. This study investigates the feasibility of utilizing TiO_2_ as a photocatalytic hydrophilicity promoter in the fabrication of composite coatings for various applications.

## 1. Introduction

Electrodeposition of composite coatings is one of the cheapest, simplest, and most versatile surface modification methods [1,2]. It is based on the introduction of inert second-phase particles into the plating electrolyte. Pure nickel coatings are used in various industrial applications due to their high microhardness, corrosion, and wear resistance. Moreover, nickel is frequently used as a metal matrix for composite coatings [3]. The introduction of the second phase particles into the Ni matrix can effectively tailor the properties of the formed composites. Previous studies demonstrated that the inclusion of 0.5–5.0 wt.% of solid particles (oxides and salts) into the nickel matrix could improve the microstructure [4,5,6], wettability [7], physicochemical [8], tribological [9,10], photocatalytic [11,12], and antibacterial [13] properties of the initial surface.

The composition of nickel-plating electrolytes, parameters of electrodeposition (pH, agitation, and current density), size, and concentration of the second phase particles are of particular importance. The Watts electrolyte is the most commonly used in industry for the deposition of Ni and Ni-based coatings. It has some advantages, such as relatively wide ranges of operating current densities and pH [14]. One of the important components of the Watts electrolyte is boric acid (H_3_BO_3_), which stabilizes the pH of the electrolyte and controls the nucleation and grain growth of Ni deposits [14,15]. However, the European Chemicals Agency has considered boric acid as a SVHC (substance of very high concern) [16]. Moreover, according to the guidelines of the World Health Organization (WHO), the concentration of boron (B) in drinking water and large desalination plants is restricted to 0.5 ppm and 0.3–0.5 ppm, respectively. It is also well-known that the boron concentration in global seawater varies between 4 and 6 ppm [17]. Irrigation water is the most critical source of boron contamination, such that prolonged irrigation with water containing >1 ppm of boron can have a detrimental effect on soil and plants [18]. However, the concentration of boric acid in the standard Watts nickel-plating electrolyte is up to 30 g/L (30,000 ppm) [14], making its utilization expensive and energy-consumptive. The removal of boron to accepted levels requires specific and expensive treatment strategies. As an example, a regular one-pass standard seawater reverse osmosis (SWRO) cannot meet the above-mentioned requirements as boron is in the non-ionic undissociated boric acid form, indicating its small molecular size due to the nearly neutral pH of seawater (7.5–8.5), which can easily penetrate through the membrane. The increase in pH before the first pass in SWRO increases the scaling formation, which in turn reduces the RO system performance and increases the capital cost (the requirement of a high dosage of antiscalant). Therefore, the two-pass design is usually necessitated to fulfill the guidelines of WHO for boron removal, in which the pH will be adjusted to around 11 between the two-pass [17]. From the above discussion, it is crucial to substitute boric acid with other effective buffer agents in the nickel electroplating industry [19].

In our previous works [20,21,22,23], we have developed a novel type of tartrate electrolytes for electrodeposition of Ni at ambient temperature and examined their properties. The proposed tartrate electrolytes do not require heating of the deposition bath, thus decreasing operation cost and excluding the step of the bath’s composition adjustment due to water evaporation. Compared to Ni coatings obtained from the Watts electrolyte, which is commonly utilized at 45–55 °C [24], coatings obtained from low-temperature electrolytes have slightly lower microhardness [20,25,26]. Such coatings can also be defined as a soft matrix, which facilitates the polishing and drilling of the inner phase of composite materials and opens up new promising exploitation opportunities for these types of Ni-based coatings, such as dental applications (dental drills) and polishing wheels [27,28].

Nanosized TiO_2_ and CeO_2_ oxides are insoluble in water and are well-known for their photocatalytic activity, anti-bacterial properties [29,30], and influence on surface wettability [31]. Therefore, modification of nickel coatings with these oxides can lead to the formation of novel composite materials with advanced functionality. To the best of the authors’ knowledge, no systematic studies devoted to comparing the properties of the above-mentioned composite coatings obtained at ambient temperature have been performed to date. A few studies have investigated the effects of Ti and Ce oxides on the properties of Ni coatings deposited at temperatures from 30 to 60 °C from plating baths based on the mixture of chlorides and sulfates (Watts electrolyte) or sulfamates (Table 1 and Table 2).

As can be seen from Table 1 and Table 2, several particle sizes and concentrations were analyzed by other researchers. Particularly, TiO_2_ and CeO_2_ particles with a mean size from 21–350 nm to 3 μm and 15–30 nm to 3 μm, respectively, were added as the second phase to nickel-plating electrolytes. The concentration of TiO_2_ in electrolytes varied between 1 and 15 g/L, whereas the variation for CeO_2_ was from 10 to 60 g/L. The majority of studies used specific concentrations of the second phase particles [32,33,34], while some others studied a range of concentrations [35,36]. The variety of examined properties has been determined by the type of the incorporated second phase. Although the same type of second phase particles has been employed in these studies, variation in the second phase concentration and particle sizes, as well as composition and conditions of electrodeposition, substantially altered the physicochemical and mechanical properties of the obtained composites (Table 1 and Table 2). Additionally, almost all studies focused only on specific properties of the composite coating. Moreover, Ni-CeO_2_ coatings were predominantly electrodeposited from electrolytes that contained organic additives/surfactants (brighteners, leveling additives) [34,37,38,39,40,41]. Such additives themselves strongly influence the microstructure and increase the microhardness of nickel matrix up to 450–600 HV [29,42]. At the same time, organic additives decrease the stability of the electrolyte and complicate the electrodeposition process. Therefore, it is preferable to use simple electrolytes with high dispersion stability for the deposition of composite coatings.

**Table 1:** Influence of TiO_2_ particles on properties of nickel matrix.

Electrolyte Type	Examined Properties						Conditions of Electrodeposition			Ref.
Watts	Corrosion	Wear	Hydrophilicity	Photocatalytic	Microroughness	Microhardness, HV	Temperature, °C	Concentration of TiO_2_, g/L	Particle Size, nm	
Watts	√	√			√	387	50	12	50	[32]
						1483	30	6	50	[43]
					√		55	10–80	200–350	[35]
Watts +surfactant		√			√	640	60 ± 5	5–15	>100	[44]
	√	√				313	45	1	50, 80	[45]
			√				50	45	25	[37]
		√					50	10–25 mL/L	–	[46]
Sulphamate +surfactant	√	√				824	50	50	2–3 μm	[47]
Sulphamate, pyrophosphate	√	√				620	40	1–10	21	[33]
Methansulfonate				√			60	1–7	30	[48]
Tartrate	√	√	√	√	√	√	22	10	~11 μm	present contribution

**Table 2:** Influence of CeO_2_ nanoparticles on properties of nickel matrix.

Electrolyte Type	Examined Properties			Conditions of Electrodeposition			Ref.
Watts	Photocatalytic	Microroughness	Microhardness, HV	Temperature, °C	Concentration of TiO_2_, g/L	Particle Size, nm	
Watts +surfactant	√		760 ± 100	45	10–50	15–20	[38,49]
		√	560	55	5–20		[39]
	√		994	55	1	20	[40]
				50	10	–	[50]
	√			25 ± 3	5		[51]
sulphamate +surfactant		√		50 ± 1	10–50	30	[52]
	√	√	508		100	22	[34]
		√		45	10–60	3 μm	[53]
Watts	√	√	824	50	5–20	15–25	[36]
acetate+H_3_BO_3_ +surfactant	√		725	30	3–12	–	[41]
Tartrate	√	√	√	22	10	~46 μm	present contribution

All hardness values reported in Table 1 and Table 2 are average of five to ten indentations at randomly selected positions. √ means properties that were studied in the publication.

Consequently, in the present contribution, we employed a simple one-step galvanostatic electrodeposition, from the low-temperature electrolyte based on Na, K-tartrate buffer agent (Rochelle salt) as a greener substitute of the traditionally used boric acid aiming to obtain new types of soft-matrix Ni, Ni-CeO_2_, and Ni-TiO_2_ coatings onto steel or copper substrates. Herein, a detailed investigation of the physicochemical, mechanical, photocatalytic characteristics, and corrosion properties of obtained composites and pure nickel coatings was conducted using various analytical techniques. This study was designed to contribute to a deeper understanding of the physicochemical properties of composite nickel coatings prepared with TiO_2_ and CeO_2_ as second-phase particles for their potential industrial use. For this purpose, several analytical methods (SEM, EDX, XRD, and XPS) were used to investigate the composition and structure of the nickel and composite coatings as well as characterize products of corrosion.

## 2. Materials and Methods

### 2.1. Materials and Conditions of Deposition

**Figure 1:**
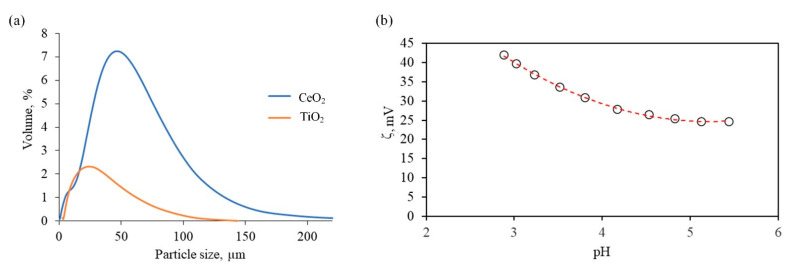
Particle size distribution for CeO_2_ and TiO_2_ (**a**) and ζ-potential versus pH for CeO_2_ (**b**).

In the electrodeposition process, copper or steel plates were used as a cathode (substrate), and Ni plates served as an anode. Prior to electrodeposition, the surface of the cathode was treated according to the procedure described in detail in our previous work [22]. Briefly, the surface of the cathode was mechanically ground with #500, #900, #1200, and #2000 emery paper, cleaned in an alkaline solution, and etched in nitric acid. Ultra-pure deionized water (DI) (Merck Millipore Q-POD, DI, 15 MΩ) was used to rinse the substrates after each step, and finally, samples were dried with N_2_ gas at ambient temperature. Electrodeposition of nickel coatings was performed from the electrolyte with composition and under conditions represented in Table 3, which were chosen based on our previous study [22]. All chemicals were used as received. The temperature of the electrolyte was controlled by an RK8 CS water bath. Electrolysis was carried out in a galvanostatic mode using a PS 3005 power supply. The pH of the solutions was monitored using a Radiometer PHM 240 pH/ion pH meter. Suspensions for electrodeposition of composite nickel-based coatings were prepared by adding either TiO_2_ or CeO_2_ particles into the electrolyte to reach oxide concentration of 10 g/L. The commercial TiO_2_ and CeO_2_ powders were characterized by XRD, Figure S1 (Supplementary material), particle size distribution was measured with a Beckman Coulter LS 13320 laser diffraction particle size analyzer in Fraunhofer optical model (Figure 1a), and zeta potential of CeO_2_ was measured by a SurPASS electro-kinetic analyzer, Figure 1b.

ζ-potential of CeO_2_ particles was measured in a cylindric cell by a SurPASS electro-kinetic analyzer using 1 mM KCl solution as the background electrolyte. A 0.05 M HCl solution was used during ζ-potential analysis to decrease the pH of the solution to 3. ζ-potential of TiO_2_ particles was not examined because the average size of particles was less than 25 μm. Finally, the zeta potential was calculated from streaming current measurement according to the classic Helmholtz–Smoluchowski equation (Equation (1)).

(1) $$\zeta =\frac{dl}{dp}\cdot \frac{\eta }{\varepsilon \cdot \varepsilon_{0}}\cdot \frac{L}{A},$$

where *dl*/*dp* is the slope of streaming current versus pressure, *η* is the liquid viscosity (viscosity of pure water at room temperature about 20 °C), *ε*_0_ is the vacuum permittivity, and *ε* is the dielectric constant of the electrolyte (water at the same temperature), *L* is the length of the streaming channel, and *A* is the cross-section of the streaming channel.

The TiO_2_ powder was predominantly in anatase phase (Figure S1) with the mean particle size (Figure 1a) of 11 μm (D_10_: 6.76; D_50_: 40.6; D_90_: 91). The CeO_2_ powder revealed distinct intense lines corresponding to the fcc structure (Figure S1). The mean size of CeO_2_ particles (Figure 1a) is 46.6 μm (D_10_: 0.31; D_50_: 1.11; D_90_: 36.1). The measured mean size varied from that provided by the manufacturer, perhaps, due to the particle’s agglomeration.

The cathode current efficiency of the nickel coating without second-phase particles was estimated to be about 95% by weight the cathode before and after electrolysis using a Mettler AC 88 analytical balance. Coatings with a thickness of about 9 μm for electrochemical and photocatalytic tests and 25–30 μm for all other tests were obtained. The coatings’ thickness was determined by examining cross-sections with a scanning electron microscope.

### 2.2. Material Characterization

After deposition, obtained composites were ultrasonically cleaned in acetone for 10 min in order to remove loosely adsorbed TiO_2_ or CeO_2_ particles from the surface. Thereafter, the coatings were rinsed with distilled water and dried in a flow of N_2_. Characterization of the surface morphology, composition, and elemental distribution over the surface was carried out using a Hitachi SU3500 scanning electron microscope (SEM, Tokyo, Japan) equipped with an energy dispersive X-ray (EDX) unit. The chemical composition and crystal structure of coatings were ascertained using X-ray diffraction (XRD) (PANalytical MPD, the Netherlands) with a cobalt X-ray tube. The 2*θ* range was 20–70° and the step size was 0.02 °/s. The grain size was calculated using the Debye-Scherrer equation (Equation (2)).

(2) $$D=\frac{0.94\lambda }{\beta \cos\theta },$$

where *D*, *λ*, *β*, and *θ* are the mean crystalline size (nm), X-ray wavelength (0.178897 nm), the full width at half maxima in intensity, and Bragg diffraction angle, respectively.

The microhardness of the coatings was measured at room temperature by indention using a Struers DuraScan 70 setup. A pyramidal diamond tip was used for the test. The indenter was lowered to the matrix with a 0.5 kg load (HV5) and then retrieved from the coating surface. A total of ten indentations were made at the center and on edges with a distance of 1 mm on each specimen surface, and the average values were calculated.

**Figure 2:**
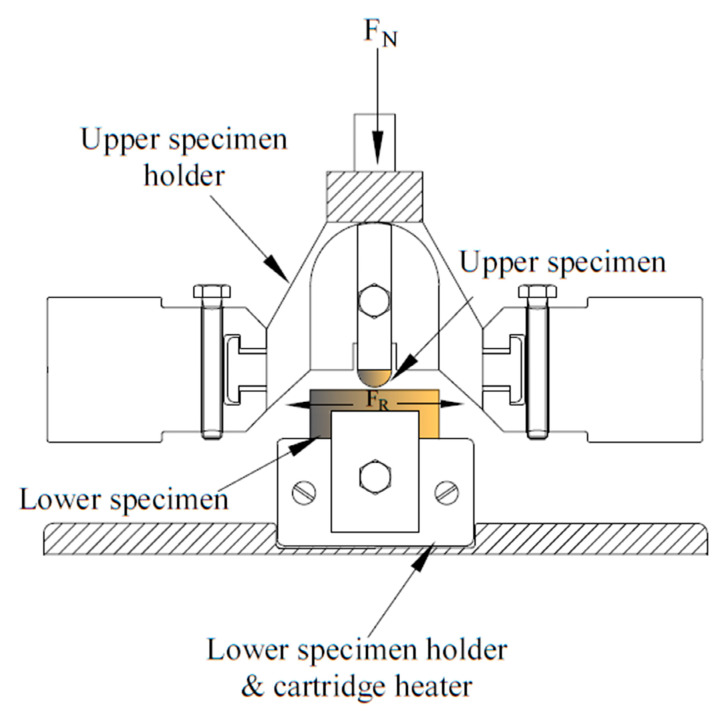
Setup for the wear measurement.

The tribological tests of nickel and composite nickel-based coatings were carried out using an Optimol SRV high-temperature reciprocating friction and wear tester in a ball-on-disc test configuration. In the wear equipment depicted in Figure 2 an upper specimen (Si3N4) is loaded against the lower stationary disc (coated specimen) and oscillated by means of an electromagnetic drive. The lower specimen is mounted on a specimen block incorporating a heating element that allows conducting tests at temperatures up to 900 °C. The data acquisition and control software enabled the measurement and control of different test parameters, such as friction coefficient, frequency, stroke length, temperature, and load. All wear tests were performed using a frequency of 50 Hz and speed of 200 mm/s in a dry sliding mode at room temperature with a load of 12 N. The stroke length was 2 mm, and the total sliding length was 180 m with the data recorded automatically during tests. After the tests, the specimen was washed with distilled water to remove the debris from the surface. The wear behavior and the wear rate of the samples were studied by making profilometric measurements of wear tracks. The wear rates were calculated based on the profiles of wear tracks vs. duration of wear and the corresponding wear scar depth and length were determined by a Zygo NewView 7300 3D optical interferometer. The volume of scars after the test was calculated using the MountainsMaps ^®^ software 7.4 (Digital Surf, Besançon, France).

Wear rate, *R*, was calculated by dividing the volume loss by the load and the total sliding distance on the disk specimen according to the following equation (Equation (3)):

(3) $$R=\frac{V}{2\times L\times S\times N},$$

where *V* is the volume loss of material during the test duration; *L* is the applied load; *S* is the stroke length; *N* is the total number of reciprocations.

The surface hydrophilicity of electrodeposited composites and pure Ni was evaluated by measuring the average equilibrium sessile drop contact angles of deionized water using a KSV CAM 101 instrument (Helsinki, Finland). Nickel and composite Ni-CeO_2_ and Ni-TiO_2_ coatings were initially cleaned with ethanol and gently dried at room temperature. Afterward, the contact angle (CA) was measured by putting a droplet of deionized water using a microsyringe (~5 μL) on the top of the examined surface. With the aid of a CCD camera (DMK 21F04, The Imaging Source Europe GmbH, Bremen, Germany), which is connected to the instrument, the image of the droplet was taken, and further treated by curve fitting analysis with the CAM 2008 software. The final reported CA results are the average of at least 8 measurements. Images were analyzed based on statistics measurement and Young/Laplace fitting methods.

The photocatalytic activity of Ni, Ni-CeO_2_, and Ni-TiO_2_ coatings was evaluated by performing methylene blue (MB, CAS No. 61-73-4, Sigma Aldrich, St. Louis, MI, USA) degradation under simulated solar light. A sample with a surface area of 15 cm^2^ was placed into 50 mL of 0.03 mM aqueous MB solution in a beaker coupled with a jacket for adjusting the temperature (20 ± 1 °C) and exposed to a simulated solar light source (Max-350 Compact Xenon Light Source, Japan) for up to 6 h. Illumination was applied to the solution and sample from the top side at a distance of ~14 cm. The intensity of the incident light was measured to be about 33 mW/cm^2^. The MB solution was initially stirred for about 2 h in darkness to reach the adsorption equilibrium. During light irradiation, the reaction mixture was continuously stirred on a magnetic stirrer, and aliquots of the reaction mixture were collected at regular time intervals. The leftover MB concentrations were estimated with the help of a UV-vis spectrophotometer (Jasco V-670 spectrophotometer, Tokyo, Japan). The maximum absorption peak (λ_max_) of MB at about 666 nm was considered to estimate the remaining concentration of MB in aqueous solutions [54,55]. All experiments were performed in triplicate to ensure the statistical reliability of the results.

Electrochemical measurements were carried out in a three-electrode thermostated cell using an Autolab PGSTAT 302N potentiostat-galvanostat (Methrom Autolab BV, the Netherlands). The measured setup contained a saturated silver/silver-chloride reference electrode, a platinum wire counter electrode, and a copper substrate with the examined coating (exposed surface area 1 cm^2^) as a working electrode. Electrochemical corrosion studies of nickel and composite nickel-based coatings were carried out in 0.5 M NaCl solution at room temperature (~20 °C–22 °C). Polarization curves were recorded at a potential sweep rate of 1 mV/s in the potential range of ±300 mV from the open circuit potential (OCP). The corrosion current density (*i*_corr_) of the specimens was determined by extrapolating the Tafel regions of anodic and cathodic branches of the polarization curves [22] and corrosion rate (*v*_corr_) is calculated according to [56]. The electrochemical impedance spectroscopy (EIS) spectra values were recorded at the OCP in the frequency range from 10 kHz to 0.01 Hz with an amplitude of 10 mV. The measured frequency responses were interpreted using a nonlinear least square fitting procedure in Nova 2.1 and ZView 3.2c software. Prior to experiments, the working electrode was immersed in the electrolyte for 30 min for OCP stabilization. All electrochemical studies reported in the present work were at least triplicated.

For weight loss tests, nickel and composite nickel-based coatings were exposed to 50 mL of 0.5 M NaCl solution for 3.5 months. Samples (4 cm^2^) were periodically removed, washed thoroughly with DI water, weighted using a microbalance, and examined by SEM. The weight differences of the specimens were recorded after 5, 10, 20 days, and 3.5 months of immersion. At the same time, a portion of corrosion media was analyzed with inductively coupled plasma mass spectroscopy (ICP-MS, an Agilent 7900) in a mixture of 1% HNO_3_ and 1% HCl (Ultrapure, Merck) to evaluate the concentration of released nickel ions. The relative standard deviation of all ICP measurements was less than 3.6%.

X-ray photoelectron spectroscopy (XPS) was used for the characterization of coatings after corrosion tests. The samples were cleaned in deionized water for 10 min and dried with N_2_. No other treatment was implemented in order to not compromise the subtle chemical changes on the surface. Spectra were recorded in the Ni2p, Ti2p, Ce3d, Cl2p, C1s, and O1s binding energy (BE) ranges for each sample to verify their surface chemistry. The measurements were carried out using an Escalab 250Xi multispectroscope (ThermoFisher Scientific) equipped with an Al *K*α monochromatic X-Ray source with a spot diameter of 0.65 mm. Applied pass energy was 10 eV, and the energy step size was 0.1 eV. Charge compensation was controlled through low-energy electron and low-energy Ar^+^ ions emission using the flood gun (emission current 0.15 mA). For the final peak calibration adventitious carbon signal C1s at 284.6 eV were used.

## 3. Results

### 3.1. Microstructure and Phase Composition of Nickel and Composite Nickel-Based Coatings

**Figure 3:**
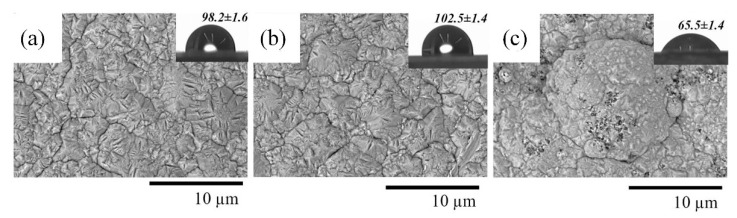
SEM images of Ni (**a**), Ni-CeO_2_ (**b**), and Ni-TiO_2_ (**c**) coatings. Insets show images of surface contact angle measurements. The contact angle of steel substrate was 79.9 ± 1.0°.

The SEM micrographs and the surface EDX composition of pure nickel and composite coatings are depicted in Figure 3 and Table 4, respectively. The nickel coating has a uniform structure with relatively straight grain boundaries of an average size of around 2–3 μm, see Figure 3a. No significant change in the surface morphology in comparison with the nickel coating was noticed due to the incorporation of CeO_2_ particles (Figure 3b), whereas the nickel-TiO_2_ composite (Figure 3c) reveals a different microstructure. The surface of composite nickel-TiO_2_ coatings has numerous nodules and spherical structures with a size of 10 μm. A similar microstructure was reported in [32,35,37] and could be explained by increasing the overpotential of nickel electrodeposition in the solutions containing TiO_2_ particles [57]. Based on the results of the EDX analysis (Table 4), nickel-TiO_2_ and nickel-CeO_2_ composite coatings have 2.1 and 0.4 wt.% of incorporated Ti and Ce, respectively.

**Figure 4:**
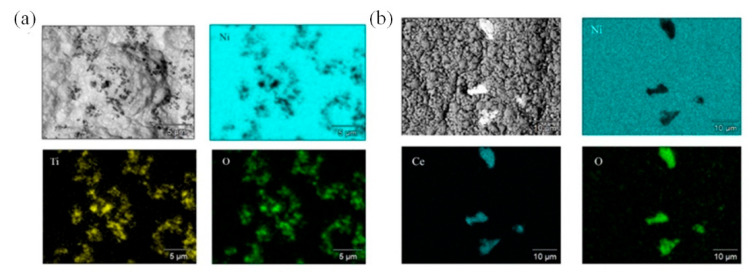
High-resolution EDX elemental mapping of the main elements in Ni-TiO_2_ (**a**) and Ni-CeO_2_ (**b**) coatings.

The contact angle (CA) measurement is frequently utilized as an index for evaluating hydrophilicity. As can be seen in Figure 4, the hydrophilicity of the Ni-TiO_2_ composite surface was increased by about 33% as compared to pure Ni, while the introduction of CeO_2_ contributed to a 4.5% increase in the CA, making the surface more hydrophobic. The contact angle (CA) for pure Ni was in good agreement with the reported CA (93–104°) obtained in previous studies [11,58], indicating a relatively hydrophobic characteristic of the pure nickel surface. Moreover, the improvement of hydrophilicity (i.e., lower contact angle) as a result of the incorporation of TiO_2_ nanoparticles into the Ni matrix in this study corroborates the earlier findings [11,58]. In the case of surface wettability behavior, both surface roughness and chemical composition play a vital role. A broad absorption band in the region of 3000–3600 cm^−1^ visible in the FTIR spectrum of pure TiO_2_ nanoparticles (Figure S2) was assigned to the hydrophilic –OH surface bonding groups and their stretching vibration on the surface of TiO_2_. In addition, the FTIR spectrum can confirm the surface-adsorbed water molecules (H–O–H bending, adsorption band at ~1626 cm^−1^) for pure TiO_2_ particles, confirming a strong interaction with water molecules. The photocatalytic activity of TiO_2_ particles, which can be activated even under visible light [59], could also be considered as another parameter that enhances the wetting ability of the Ni-TiO_2_ composite surface. During the photocatalytic action of TiO_2_, an excited state occurs in which the electron from the valence band is excited to the conduction band, and thus the Ti(IV) state reduces to the Ti(III) state. Created oxygen vacancies due to this excitation can be further occupied by water molecules, producing surface adsorbed OH radical groups and, thus, a more hydrophilic surface [59]. In general, the hydrophilicity mechanism on the Ni-TiO_2_ composite surface seems to be governed by the titania nanoparticles’ efficient dispersion/immobilization, chemical composition (presence of hydrophilic functional group), and their photocatalytic activity.

Generally, rare-earth oxides (REOs) have hydrophobic nature as they have a lower tendency to exchange electrons and form a hydrogen bond with interfacial water molecules, and, thus, they have been considered an effective way of making superhydrophobic surfaces [60,61]. Similar to the current study, the incorporation of CeO_2_ into Ni-CeO_2_ composite coatings by electrodeposition leads to the formation of a hydrophobic surface, as previously reported [51,61,62].

The incorporation of CeO_2_ and TiO_2_ into the Ni matrix is also illustrated by the EDX mapping results presented in Figure 4.

**Figure 5:**
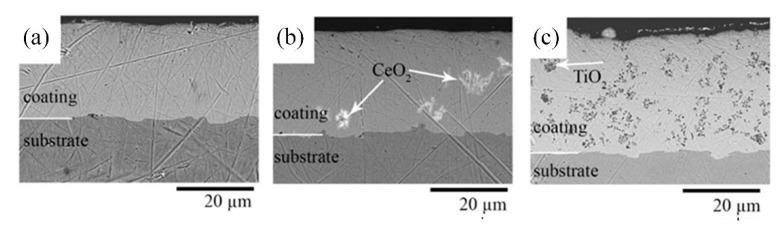
Cross-sectional SEM images of Ni (**a**), Ni-CeO_2_ (**b**), and Ni-TiO_2_ (**c**) coatings.

Ni-TiO_2_ composite coatings are characterized by an almost uniform distribution of TiO_2_ in the Ni matrix. However, the CeO_2_ particles are not homogeneously spread in the nickel matrix, and that could be the reason for the lower influence of these particles on the morphological and structural changes in the volume of the coating (Figure 3). On the other hand, the cross-section SEM images (Figure 5) show that particles are not simply adsorbed on the surface but are distributed in the bulk of the coating. Scratches seen in the SEM cross-section images originate from the polishing procedure.

The uniform structure without visible cracks at the interface between the coating and the substrate was observed. Such a homogeneous distribution of the TiO_2_ particles in the nickel matrix is expected to enhance the physicochemical properties of the formed composite coating.

**Figure 6:**
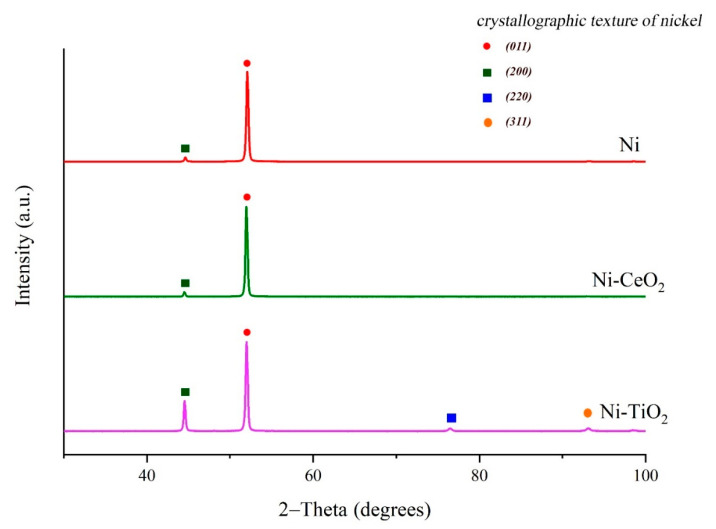
XRD patterns of Ni and composite Ni-CeO_2_ and Ni-TiO_2_ coatings.

XRD patterns of pure Ni, Ni-CeO_2_, and Ni-TiO_2_ composites are presented in Figure 6. The XRD pattern of the Ni coating (Figure 6) shows the main peaks at 44.1° and 52.2°, which correspond to the nickel (111) and (200) crystal planes typical for the fcc structure. The main peaks in the XRD pattern of the Ni-TiO_2_ coating are shifted compared to the standard XRD pattern of nickel (111) plane at 44.51° and (200) plane at 51.84° (JCPDS No. 04-0850), the 2θ of the crystal planes is slightly shifted to lower angles due to the lattice distortion as a result of TiO_2_ particles in the nickel matrix composites [33]. XRD patterns of Ni and Ni-CeO_2_ coatings overlap each other, indicating that the inclusion of CeO_2_ particles into the coating does not change the crystallographic structure of the matrix. A possible explanation could be attributed to the small number of incorporated CeO_2_ (Table 4), which possesses a larger mean size (~46 μm) compared to TiO_2_. For Ni-TiO_2_ coating, the increase of (111), (220), and (311) planes’ intensity in the face-centered cubic lattice of Ni was determined. A similar trend was also reported by Thiemig and Bund [33]. In the electrodeposition process, the presence of TiO_2_ and CeO_2_ particles as nucleation sites could promote the formation of new grains and inhibit the growth of the already formed grains. According to Debye-Scherrer’s Equation (2), the size of the crystallites calculated for the Ni (111) plane was 18–45 nm.

### 3.2. Physicochemical Properties

The surface properties of coatings, such as microhardness and microroughness, which have critical effects on the protective performance, are presented in Table 5. As can be seen, no significant change in the surface roughness *(S*_a_ = 0.26 µm) was observed when the CeO_2_ was incorporated into the Ni matrix, whilst it increased by about three times for the Ni-TiO_2_ coating. This result is in line with the study of Stanković et al. [35], who reported a substantial increase in microroughness in the presence of TiO_2_. The microroughness of nickel coatings deposited from tartrate electrolyte is ca. 2 times lower compared to Ni coatings deposited from the Watts electrolyte.

As shown in Table 5, the inclusion of CeO_2_ and TiO_2_ particles in the nickel matrix improved the microhardness by ~20% and ~40%, respectively. The increase in measured hardness of the composite coating containing CeO_2_ and TiO_2_ is usually attributed to the Orowan dispersion hardening mechanism [36,64,65]. However, in the examined case, the particle size of the used CeO_2_ and TiO_2_ was much larger than the grain size of the Ni matrix. Therefore, the impact of the Orowan hardening can be neglected. As the crystallite sizes of the Ni matrix of these two coatings are similar, the rule of the mixture has the main impact in the microhardness [66]. In this case, the change of microhardness is mainly attributed to the fraction of CeO_2_ and TiO_2_ particles embedded in the metallic matrix, and it can also be affected by the current density and the deposition time [25]. Compared to coatings from the standard Watts electrolyte (269.46 ± 12 [14]), and the Watts electrolyte that operates at ambient temperature (201 HV [67]), Ni coatings obtained in the present contribution have lower microhardness (191 ± 10 HV), which could also be caused by significant differences in pH of the electrolyte [14]. Here it is important to mention that we based the comparison on the experimental results provided by references [14] and [67] not in a sense of comparable electrolyte parameters, but as a reference of the absolute standard that is usually in use in the industry.

**Figure 7:**
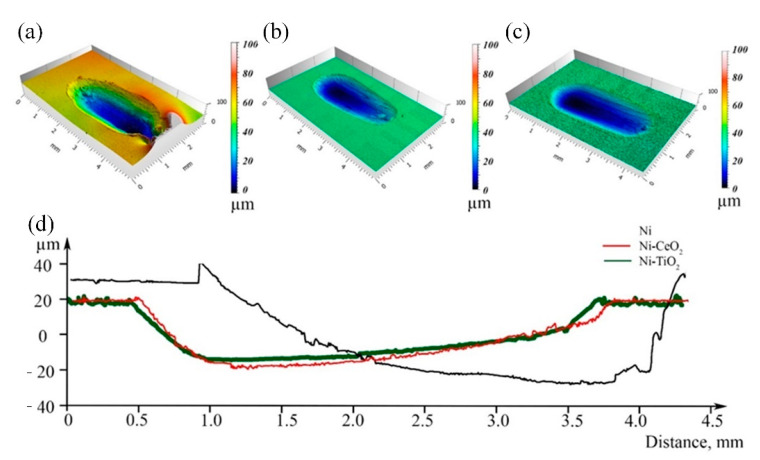
3D optical surface profile images of Ni (**a**), Ni-CeO_2_ (**b**), and Ni-TiO_2_ (**c**) coatings after wear tests and corresponding profiles of the wear tracks (**d**).

The profiles of wear tracks on the surface of the obtained nickel and composite coatings are presented in Figure 7. The wear track of the Ni coating, Figure 7a, is far wider than that of the composites (Figure 7b,c). Moreover, cracking and spalling on the worn surface of the pure Ni coating can be observed. This shows that without incorporating CeO_2_ and TiO_2_ particles into the Ni matrix, its wear resistance is significantly lower.

In the case of the co-deposition of nickel with TiO_2_ (Figure 7b) and CeO_2_ (Figure 7c), the depth of the wear tracks significantly decreased. This indicates that the incorporation of CeO_2_ and TiO_2_ particles in the Ni matrix can largely reduce the wear of the nickel-based composite coatings. Besides the clear reduction in wear depth, no plastic deformation was observed for either the coatings with CeO_2_ or TiO_2_, whereas the Ni coating was severely deformed. No significant differences were observed concerning the wear resistance based on the type of particles used, TiO_2_ or CeO_2_. The largest difference between the two types of coatings was associated with the surface roughness after the deposition, which was highest when TiO_2_ was used. The composite with embedded CeO_2_ particles had a similar surface roughness compared to the pure Ni coating.

### 3.3. Corrosion Properties

#### 3.3.1. Potentiodynamic and EIS Measurements

**Figure 8:**
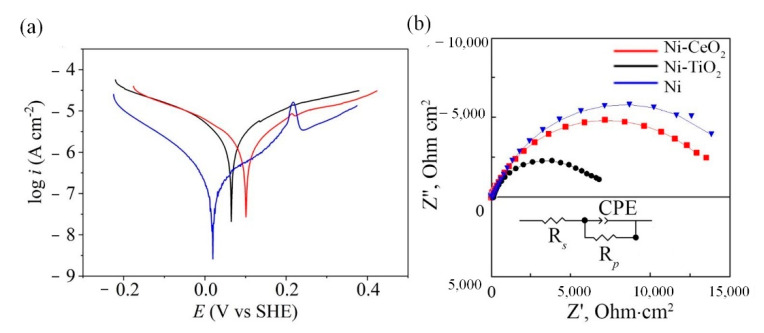
Potentiodynamic polarization curves (**a**) and Nyquist EIS plots (**b**) of Ni, Ni-TiO_2_, and Ni-CeO_2_ coatings in 0.5 M NaCl solution and equivalent circuit, used for EIS data analyses.

Figure 8 shows potentiodynamic polarization curves (a) and Nyquist EIS plots (b) of pure Ni, Ni-CeO_2_, and Ni-TiO_2_ composite coatings. The main electrochemical parameters were extracted from the potentiodynamic polarization curves and are represented in Table 6. The corrosion current density *i*_corr_ and the Tafel slopes *b*_c_ and *b*_a_ were extracted from the Tafel region of polarization curves.

The corrosion potential (*E*_corr_) of Ni-CeO_2_ and Ni-TiO_2_ composite coatings shifts toward more positive potentials compared to Ni by the values of 81 and 47 mV, respectively (Table 6). The introduced heterogeneities are the primary reason for worsening the corrosion resistance, as indicated by the values of the corrosion current density *i*_corr_ (Table 5). Surprisingly, there was no reinforcement of the nickel matrix, and the included particles did not act as a physical barrier to the propagation of defects [68]. On the contrary, the presence of the CeO_2_ and TiO_2_ particles in the metallic matrix formed galvanic microcells, which increased the dissolution of the nickel matrix, resulting in the initiation of local corrosion. Similar worsening of corrosion properties of coating in presence of TiO_2_ were discussed by Kasach et al. [69]. For Ni-CeO_2_ coating, which revealed almost similar corrosion resistance and degradation mechanisms as pure Ni in corrosion tests in 0.5 M NaCl solution, the air entrapped in the structures can also prevent the hydrophobic composite coating from being wetted by the corrosion medium through the limited solid contact area, and thus lower the corrosion rate [62]. This tendency can be clearly noticed between Ni-CeO_2_ and Ni-TiO_2_ composite coatings. Various coating formulations containing CeO_2_ particles have shown inhibitory and self-healing properties because OH– ions released during corrosion interact with CeO_2_ particles and formed stable hydrated CeO_2_ [70,71]. In a Ni composite matrix, CeO_2_ could therefore prevent corrosion propagation by blocking the electrolyte’s diffusion paths.

The lowest *i*_corr_ and respectively the lowest corrosion rate *v*_corr_ among the studied coatings was shown by pure nickel coating, 0.07 ± 0.072 μA/cm^2^ and 0.75 μm/year, respectively. The difference in values of the corrosion current density indicates that in the 0.5 M NaCl solution, the pure nickel coating has higher corrosion resistance than composite coatings. Nevertheless, these values are of the same order of magnitude, indicating that the surface resistance is not compromised dramatically. The low corrosion rate (0.00075–0.0032 mm/year) of the obtained coatings shows their high corrosion resistance in a commonly used NaCl media.

Similar conclusions can be made from the analysis of the EIS data (Figure 7b and Table 7). For all examined coatings, Nyquist plots obtained in 0.5 M NaCl solution have the shape of a distorted semicircle. Both Ni-CeO_2_ and Ni-TiO_2_ composites are characterized by smaller impedance responses as compared to Ni coating (see Figure 7b). This indicates lower corrosion resistance of the composite coatings. The equivalent circuit used for spectra fitting is shown as an inset in Figure 7b. The equivalent circuit includes solution resistance (*R*_s_), polarization resistance *R*_p,_ and the capacitive response of the Ni coating described by a constant phase element (CPE) [72]. The impedance of the CPE element can be calculated based on the following equation (Equation (4)):

(4) $$Z_{CPE}=\frac{1}{Y_{0}{\left(j\omega \right)}^{n}}$$

where *Y*_0_ is the CPE constant; *n* (0 ≤ *n* ≤ 1) is the mathematical factor showing the deviation from the ideal capacitive impedance response; *j* is the imaginary unit; ω is an angular frequency, rad^−1^. The CPE shows ideal capacitive behavior when its exponent *n* = 1, while the value of *n* below 1 corresponds to the degree of surface inhomogeneity. The equivalent circuit showed a good fit for all impedance plots (Figure 8b). The highest value of *R*_p_ was observed for the pure nickel coating, which can be ascribed to the smaller number of surface defects and pores on the surface due to the lack of galvanic microcells. The lowest corrosion resistance was shown for Ni-TiO_2_ coatings, for which the values of *R*_p_ were almost two times lower than those for Ni and Ni-CeO_2_ coatings. Moreover, the CPE constant *Y*_1_ fitted for Ni-TiO_2_ coating had the largest value, indicating lower capacitance of the double layer and decreased corrosion resistance. The corrosion behavior of a coating is usually closely related to its hydrophilic properties, such that the increase in hydrophilicity leads to a decrease in corrosion resistance [69]. As expected, the Ni-TiO_2_ composite coating revealed weaker corrosion resistance due to its higher hydrophilic characteristics, which increase the exposure area to aggressive media compared to the other composites.

**Figure 9:**
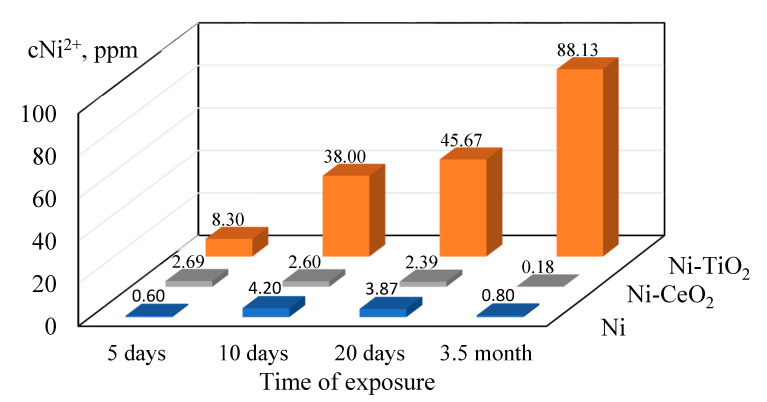
Concentration of nickel ions released into the 0.5 M NaCl solution during exposure for 3.5 months.

The long-term corrosion behavior of the studied coatings was examined by weight loss. The concentration of nickel ions released during 3.5 months of exposure to 0.5 M NaCl solution with an initial pH of 5.4 is depicted in Figure 9 and Table 8, respectively. Increasing the pH during the dissolution of nickel can be described by the mechanism explained in our previous study [72], which includes the following steps: Ni→NiOH_ads_→NiOH^+^. In the last step, hydroxide ions will be released according to the reaction as follows (Equation (5)):

NiOH^+^→Ni^2+^ + OH^−^ (5)

The highest amount of Ni^2+^ ions (~88 ppm) was released into a 0.5 M NaCl solution after 3.5 months of immersion of the Ni-TiO_2_ composite coating, which is in accordance with the polarization and EIS data. For Ni and Ni-CeO_2_ coatings, the concentration of released ions was 3.8 ppm and 2.3 ppm, respectively, after 20 days of testing in 0.5 M NaCl and then decreased to about 0.79 ppm and 0.18 ppm, respectively. The possible formation of nickel hydroxide in the bulk solution could be considered as an explanation for the changes in pH of the solution. The lower corrosion resistance of composites could be related to the pores and microcracks formed in the coatings during the deposition process and subsequent drying. The possible interconnection of the pores and microcracks might extend from the surface to the bulk of the material and, therefore, decrease corrosion resistance [73].

#### 3.3.2. Surface Characterization of Coatings after Corrosion

**Figure 10:**
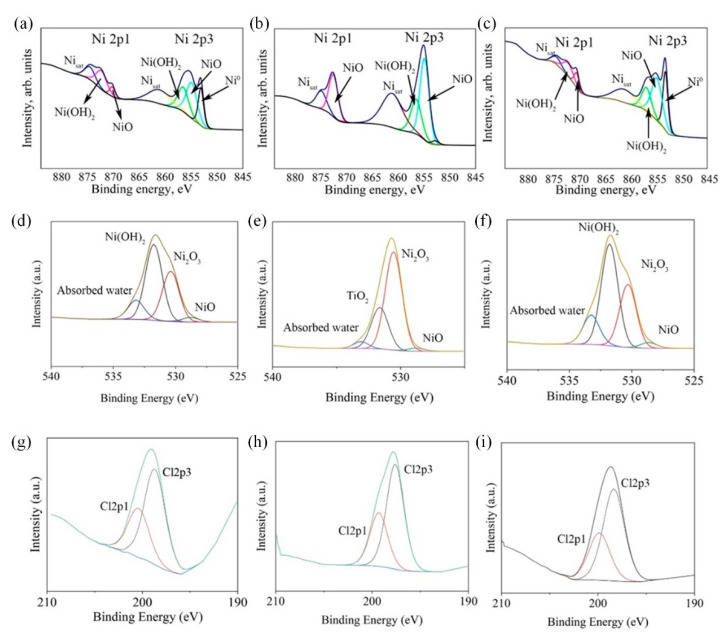
High-resolution XPS spectra for Ni2p (**a**–**c**), O1s (**d**–**f**) and Cl2p (**g**–**i**) registered after 3.5 month of immersion in 0.5 M NaCl: (**a**,**d**,**g**) Ni, (**b**,**e**,**h**) Ni-TiO_2_, (**c**,**f**,**i**) Ni-CeO_2_.

To ascertain the surface states of Ni, Ni-CeO_2_, and Ni-TiO_2_ composite coatings after immersion in 0.5 M NaCl for 3.5 months, high-resolution XPS spectra were obtained (Figure 10). Peaks corresponding to the binding energy states of Ni2p, O1s, and Cl2p were analyzed. The complex spectrum of Ni2p3/2 was deconvoluted into peaks at 851.26 eV and 854.53 eV, respectively attributed to Ni^0^ and oxidized surface Ni atoms (NiO_x_) [72], as well as the peak ascribed to Ni(OH)_2_, which exhibits at ∼857 eV [72,74]. Finally, a wide Ni2p3/2 Ni^2+^ satellite feature is present in the 860–865 eV range. O1s peak was deconvoluted into 4 peaks. Two main components, peaking at 530.2 and 531.7 eV, are characteristic of Ni oxides and hydroxides, respectively. It is worth mentioning that the Ni-TiO_2_ spectral shape is significantly different compared to Ni and Ni-CeO_2_ and has a dominant feature at 531.6 eV that can be attributed to TiO_2_ [75]. There is different oxide chemistry which might be connected with corrosion resistance. The formation of oxidized forms of nickel has been reported in previous studies for Ni-TiO_2_ [76] and Ni-CeO_2_ [77] surfaces.

Compared with Ni, the Ni-TiO_2_ coating in particular reveals altered surface chemistry, with a significantly higher share of oxidized nickel. This difference is confirmed by the eminent presence of Ni_2_O_3_ peak at both Ni2p and O1s spectra (Figure 10b,e, respectively). On the other hand, the surface chemistry registered for Ni-CeO_2_ coating is much alike Ni, which may indicate similar corrosion resistance and degradation mechanisms under 0.5 M NaCl immersion and is confirmed by the higher value of *R*_p_ obtained by impedance spectroscopy (Figure 8b). This observation is confirmed by the presence of chlorides in the layer of corrosion products [78], which were detected at ∼197.6–198 eV. The analysis showed that the amount of Cl^−^ at the surface of studied samples changes in the following ascending order: Ni > Ni-CeO_2_ > Ni-TiO_2_, Figure 10. During corrosion examinations, localized interaction of chloride ions with the nickel matrix of the deposited coatings can cause the breakdown of the surface passivity. Afterward, localized corrosion attack of the substrate in the form of pitting will occur. The most probable mechanism of the corrosion attack in this case includes two stages [79]. At first, chloride ions adsorb on the outer surface of the Ni matrix by competitive adsorption with hydroxyl ions. Further, chloride ions intersect on the metal surface, most probably by the place exchange mechanism.

**Figure 11:**
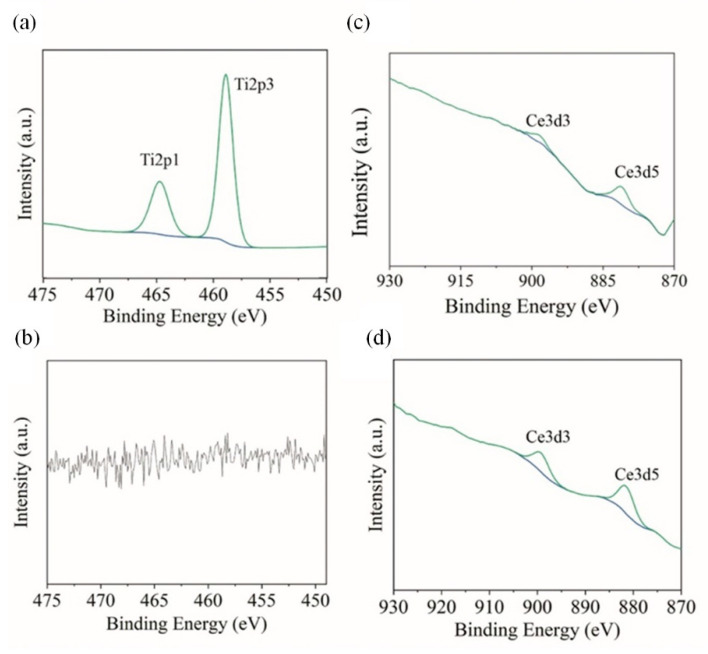
High-resolution XPS spectra for Ti2p3 (**a**,**b**) and Ce3d5 (**c**,**d**) registered before corrosion tests (**a**,**c**) and after 3.5 month of immersion in 0.5 M NaCl (**b**,**d**).

Next, the XPS spectra of Ti2p (Ni-TiO_2_) and Ce3d (Ni-CeO_2_) for the studied coatings before and after corrosion tests are depicted in Figure 11, together with the proposed deconvolution model. The Ti2p3/2 peak at 458.91 eV and Ce3d5/2 at 881.42 eV are located at the energies often found in the literature for TiO_2_ powder [80] and CeO_2_ powder [81], while metallic Ti and metallic Ce are expected to have XPS peaks at binding energies of 453.7 eV (Ti2p3/2) [82] and 885.01 eV (Ce3d3/2) [83], respectively.

Interestingly, as a result of the exposure, the signal from TiO_2_ disappears as the species are dissolved throughout the corrosion test, increasing the surface sites active to corrosion. No such observation is performed for Ni-CeO_2_ coating, where the chemistry of Ce species is intact at the end of the experiment, corroborating high corrosion resistance in electrochemical studies. The composition of Ni, Ni-CeO_2,_ and Ni-TiO_2_ composite coatings surface after immersing in 0.5 M NaCl after 3.5 months is presented in Table 9.

**Figure 12:**
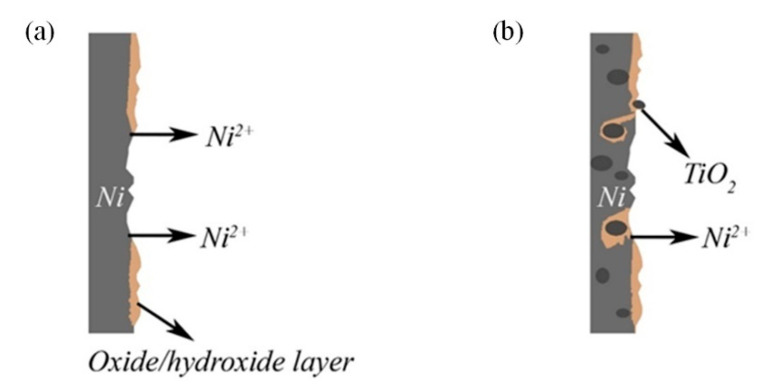
Scheme of dissolution (**a**) Ni and Ni-CeO_2_ coatings, (**b**) Ni-TiO_2_.

Long-term immersion of coatings led to the formation of a nickel oxide/hydroxide layer, which is 70.1 at.% and 76 at.% for Ni-CeO_2_ and Ni coating, respectively. In turn, the Ni-TiO_2_ coating was almost fully covered with a passive oxide/hydroxide layer (97.6 at.%), indicating the higher tendency of the Ni-TiO_2_ surface to oxidation/corrosion and explaining a small amount of metallic nickel (2.4 at.%), which might be related to their lower corrosion resistance as indicated by the impedance spectra. It is also supported by the highest release of nickel ions during immersion in 0.5 M NaCl (Figure 9). For Ni-TiO_2_ composite coatings, TiO_2_ in galvanic coupled with the Ni matrix acts as a cathode and enhances the dissolution and release of nickel ions. Note that in the case of Ni-CeO_2_ composites, the amount of the second-phase particles embedded in the matrix was much smaller, resulting in lower local galvanic activity. The dissolution of nickel leads to the mechanical removal of TiO_2_ particles (Table 9, Figure 12).

Furthermore, elemental analysis of the surface of Ni, Ni-CeO_2_, and Ni-TiO_2_ coatings (Figure 10h) showed 0.22, 0.61, and 0.82% of chloride ions, respectively. Compared to pure Ni and Ni-CeO_2_, the Ni-TiO_2_ coating revealed 3–4 times more adsorption affinity towards Cl^−^ ions when it was exposed to the NaCl medium. A possible explanation for the higher adsorption affinity of the Ni-TiO_2_ composite towards Cl^−^ ions could be related to the presence of specific potential adsorption sites (i.e., Ti and O) on the surface of TiO_2_ [80], which could preferably allow Cl^−^ to be attached to the bridging O on the surface [81].

**Figure 13:**
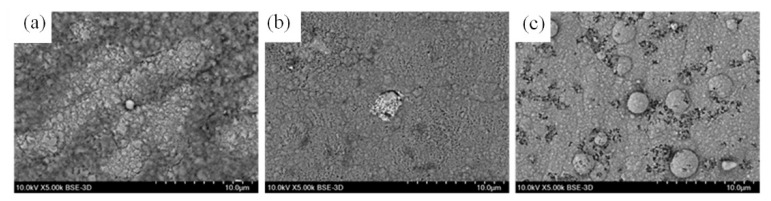
SEM images of Ni (**a**), Ni-CeO_2_ (**b**), and Ni-TiO_2_ (**c**) coatings after 3.5 months of exposure to 0.5 M NaCl.

Surface images of pure nickel and composite coatings after 3.5 months of exposure to 0.5 M NaCl are depicted in Figure 13.

A slight change in the microstructure of Ni and composite coatings after the corrosion test is clearly visible compared to the initial surface (Figure 3). For pure Ni, uniform corrosion was observed. In the case of composite coatings, corrosion is preferentially initiated at the interfaces between TiO_2_ and CeO_2_ particles and the Ni matrix. The pitting corrosion can be clearly seen in the case of Ni-CeO_2_. It can also be noticed from the SEM micrographs that Ni coatings demonstrated better corrosion resistance compared to the composites, which is in line with the XPS results.

### 3.4. Photocatalytic Activity

The photocatalytic activity of Ni and composite Ni-TiO_2_ and Ni-CeO_2_ coatings was evaluated by measuring the degradation rate of methylene blue (MB) in the aqueous solution. During irradiation, samples were analyzed with a UV–visible spectrophotometer. The mechanism of photocatalytic activity of coatings is explained in Figure S3 and can be explained by the positive holes (h+) and negative electrons (e−) formed on the TiO_2_ photo-catalyst surface due to the excitation of electrons from the valence band to the conduction band [84,85,86]. As a result, hydroxyl radicals could be produced due to the reaction of the hole with water or hydroxyl ions. The electron in the conduction band is responsible for the reduction of molecular oxygen to superoxide anion. In general, three forms of reactive species, namely, hydrogen peroxides (HO_2_∙), hydroxyl (HO∙), and superoxide (∙O^2−^), are considered the main oxidizers of the organic compounds, which are adsorbed on the oxide surface [86,87,88]. In most cases, these generated radicals react with a dye, forming radicals and radical cations, which then mineralize into carbon dioxide, water, and inorganic nitrogen with nitrate ions [86].

**Figure 14:**
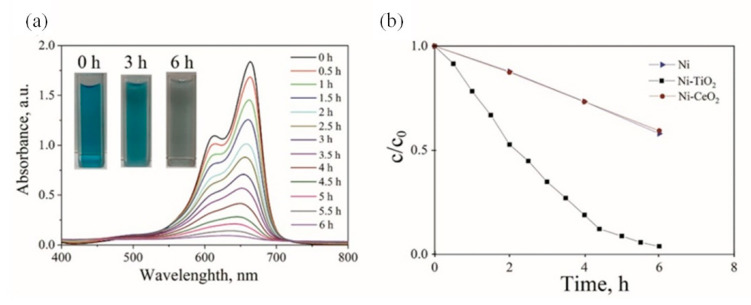
UV-Vis spectra of Ni-TiO_2_ in MB aqueous solution (**a**); Photocatalytic degradation of MB at Ni, Ni-CeO_2_, Ni-TiO_2_ (**b**).

The influence of irradiation time (6 h) of Ni-TiO_2_ composite coatings on the photodegradation of MB using visible-light radiation is presented in Figure 14. Photodegradation of MB by Ni and Ni-CeO_2_ coatings is depicted in Figure 14b and Figure S4.

The decomposition of MB on the Ni-TiO_2_ composite coating was 96.2% after 6 h of illumination. The decomposition occurred effectively on TiO_2_ particles and was related to the high crystalline anatase phase structure of TiO_2_ (Figure S1). The Ni and Ni-CeO_2_ coatings showed similar behavior in the degradation of MB after 6 h, which was around 40%.

## 4. Conclusions

This study was set out to evaluate the deposition of Ni-TiO_2_ and Ni-CeO_2_ composite coatings by the conventional direct current electrodeposition process at ambient temperature.

- The electrodeposition of pure nickel with a current efficiency of approximately 97% was performed in a simple one-step galvanostatic deposition regime from the solution based on a green buffer agent (Rochelle salt) compared to a traditionally used solution with boric acid;
- The incorporation of CeO_2_ and TiO_2_ particles in the Ni matrix increase the wear resistance of the nickel-based composite coatings by 2 times and microhardness by 1.2 and 1.4 times, respectively. The results of this investigation showed that composites possess multifunctional properties that can open new promising exploitation opportunities for these types of materials;
- Corrosion of pure nickel, as well as Ni-CeO_2_ and Ni-TiO_2_ composites, were studied by polarization and EIS techniques. Corrosion current density of 0.07 ± 0.072, 0.23 ± 0.095, and 0.29 ± 0.009 μA/cm^2^ were obtained for Ni, Ni-CeO_2_, and Ni-TiO_2_, respectively; thus, the lowest *i*_corr_ was shown by the pure nickel coating. The presence of the layer of corrosion products in the form of nickel oxides/hydroxides on the surface of coatings was proved by XPS analysis;
- Significant decomposition of methylene blue for Ni-TiO_2_ composite coating in aqueous solution after 6 h of UV light irradiation demonstrated the outstanding photocatalytic activity of this composite. Moreover, the introduction of the TiO_2_ particles into the plating solution resulted in an increase in the microroughness of the coating.

It is anticipated that current findings can be extended to other metals to synthesize coatings with multifunctional properties.

## Figures and Tables

**Table 3:** Chemicals and conditions of electrolysis.

Chemical/Parameter	Value	Purity	Suppliers
NiSO_4_·7H_2_O	0.8 mol/L	99.0%	Sigma-Aldrich
NiCl_2_·6H_2_O	0.2 mol/L	99.9%	Sigma-Aldrich
KNaC_4_H_4_O_6_·4H_2_O.	0.2 mol/L	99.0%	Sigma-Aldrich
CeO_2_	10 g/L	≥99.0%	Sigma-Aldrich
TiO_2_	10 g/L	≥99.5%	Sigma-Aldrich
Stirring Rate	400 rpm		
Current Density	200 A/m^2^		
pH	3 ± 0.1		
Temperature	22 ± 1 °C		

**Table 4:** Composition of nickel and composite nickel-based coatings based on the results of EDX analysis.

Element, wt.%	Ni	Ni-CeO_2_	Ni-TiO_2_
Ni	100	98.9	96.7
O	−	0.7	1.2
Ce	−	0.4	−
Ti	−	−	2.1

**Table 5:** Microhardness, microroughness, and wear rate of nickel and composite nickel-based coatings.

Type of Coating	Ni	Ni-CeO_2_	Ni-TiO_2_	Ni (Watts Electrolyte)
Microhardness, HV	191 ± 10	230 ± 15	270 ± 5	270 ± 12 [14]
Microroughness *S*_a_, μm	0.26 ± 0.09	0.26 ± 0.02	0.86 ± 0.03	0.41 [63]
Specific wear rate × 10^14^, m^3^/m	4.12	2.58	2.01	50–80 [14]

**Table 6:** Electrochemical parameters extracted from potentiodynamic polarization curves.

Coating	|*b*_c_|, V/dec	*b*_a_ V/dec	*i*_corr_, μA/cm^2^	*v*_corr_, mm/year	*E*_corr_, V
Ni	0.0229	0.0354	0.07 ± 0.072	0.00075	0.019 ± 0.003
Ni-CeO_2_	0.0216	0.0326	0.23 ± 0.095	0.00248	0.101 ± 0.023
Ni-TiO_2_	0.0258	0.0234	0.29 ± 0.009	0.00313	0.066 ± 0.015

**Table 7:** Parameters obtained from the fitting of the impedance spectra after exposure to 0.5 M NaCl solution.

	Type of Coating		
	Ni	Ni-TiO_2_	Ni-CeO_2_
*R*_s_, Ohm∙cm^2^	65.32	51.62	54.50
*Y*_1_, s^n^/ (Ohm∙cm^2^)	1.19 × 10^−5^	1.50 × 10^−4^	1.03 × 10^−5^
*n*_1_	0.79	0.74	0.74
*R*_p_, k Ohm∙cm^2^	16.00	6.90	14.41

**Table 8:** pH of 0.5 M NaCl solution * during 3.5 months of immersion of Ni, Ni-TiO_2_, and Ni-CeO_2_.

Time of Immersion	Type of Coating		
	Ni	Ni-TiO_2_	Ni-CeO_2_
5 days	6.6	6.6	6.5
10 days	6.9	7.1	7.2
20 days	7.2	7.4	7.2
3.5 month	7.6	7.5	8.1

* initial pH of 0.5 M NaCl was 5.4.

**Table 9:** Composition (at.%) of Ni, Ni-CeO_2_ and Ni-TiO_2_ composite coatings after immersing in 0.5 M NaCl for 3.5 months by XPS analysis.

Coating	Composition (at.%)				
	Ni^0^	NiOx	Ni(OH)_2_	TiO_2_	CeO_2_
Ni	24.0	45.2	30.8	−	−
Ni-TiO_2_	2.4	64.3	33.3	0	−
Ni-CeO_2_	29.8	47.0	23.1	−	0.5

## Supplementary Materials

The following supporting information can be downloaded at: https://www.mdpi.com/article/10.3390/ma15165550/s1, Figure S1: XRD patterns of TiO_2_ (PDF 00-064-0863 TiO_2_ Anatase, syn) and CeO_2_ powder; Figure S2: FTIR spectrum of TiO_2_ particles; Figure S3: Mechanisms of photocatalysis (a) and energy of band gap (b); Figure S4: UV-Vis spectra of (a) Ni, (b) Ni-CeO_2_ in MB aqueous solution; Table S1: Positions of XPS peaks obtained after 3.5 month corrosion tests in NaCl media. References [89,90,91,92,93] are cited in the supplementary materials.
