# Supplementary material for: Influence of CeO2 and TiO2 Particles on Physicochemical Properties of Composite Nickel Coatings Electrodeposited at Ambient Temperature
Source: Materials (Basel). 2022 Aug 12;15(16):5550. doi: 10.3390/ma15165550 (PMC9414914; doi:10.3390/ma15165550)
Supplement: Supplementary file 1 [file materials-15-05550-s001.zip › materials-1799652-supplementary.pdf]

Supplementary materials

Influence of CeO_2 and TiO_2 particles on physicochemical properties of composite nickel coatings electrodeposited at ambient temperature

Iryna Makarava[1,2,], Mohammadamin Esmaeili[1], Dzmitry S. Kharytonau[3, 4], Leonardo Pelcastre[5], Jacek Ryl[6], Mohammad Reza Bilesan[1], Esa Vuorinen[7], Eveliina Repo[1]*

1 Department of Separation Science, School of Engineering Science, LUT University, Yliopistonkatu 34, FI-53850, Finland
2 , Helmholtz-Zentrum Dresden-Rossendorf, Helmholtz Institute Freiberg for Resource Technology, Freiberg, Germany
3 Soft Matter Nanostructures Group, Jerzy Haber Institute of Catalysis and Surface Chemistry, Polish Academy of Sciences, Niezapominajek 8, PL-30239 Krakow
4 Division of Machine Elements, Luleå University of Technology, Regnbågsallén, SE-97187, Luleå, Sweden
5 Institute of Nanotechnology and Materials Engineering, Faculty of Applied Physics and Mathematics, Gdansk University of Technology, Narutowicza st. 11/12 Gdansk, PL-80233, Poland
6 Division of Materials Science, Luleå University of Technology, Regnbågsallén, SE-97187 Luleå, Sweden

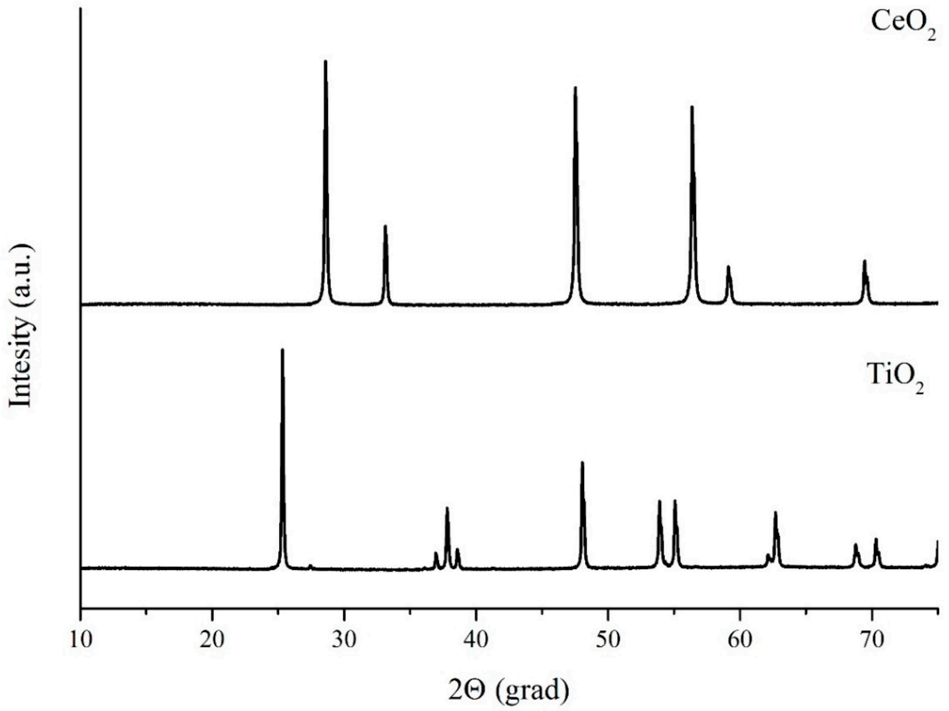

Figure S1. XRD patterns of TiO_2 (PDF 00-064-0863 TiO_2 Anatase, syn) and CeO_2 powder (PDF 00-034-0394 CeO_2 Cerianite-(Ce), syn)

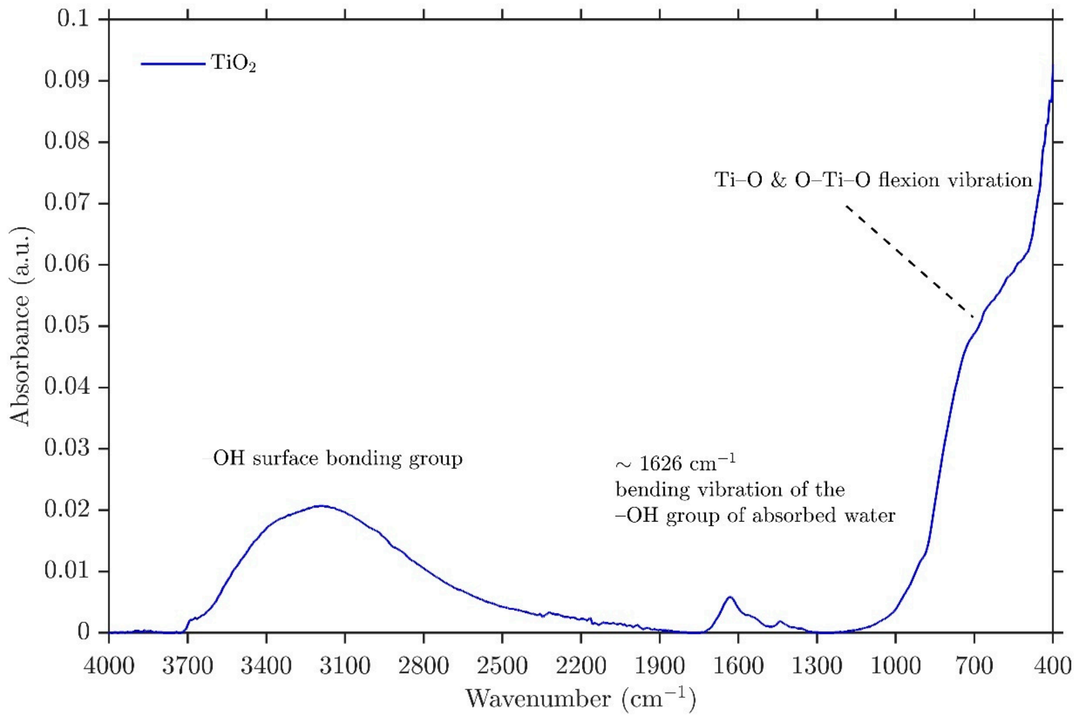

Figure S2. FTIR spectrum of TiO_2 particles

Among the well-known photocatalysts, TiO_2-containing materials have been extensively investigated for diverse catalytic applications (e.g., CO_2 reduction [1], water splitting [2], organic transformation [3], and waste decomposition [4]), due to the intrinsic merits of non-toxicity, high activity, high stability, low-price, and low environmental impact.

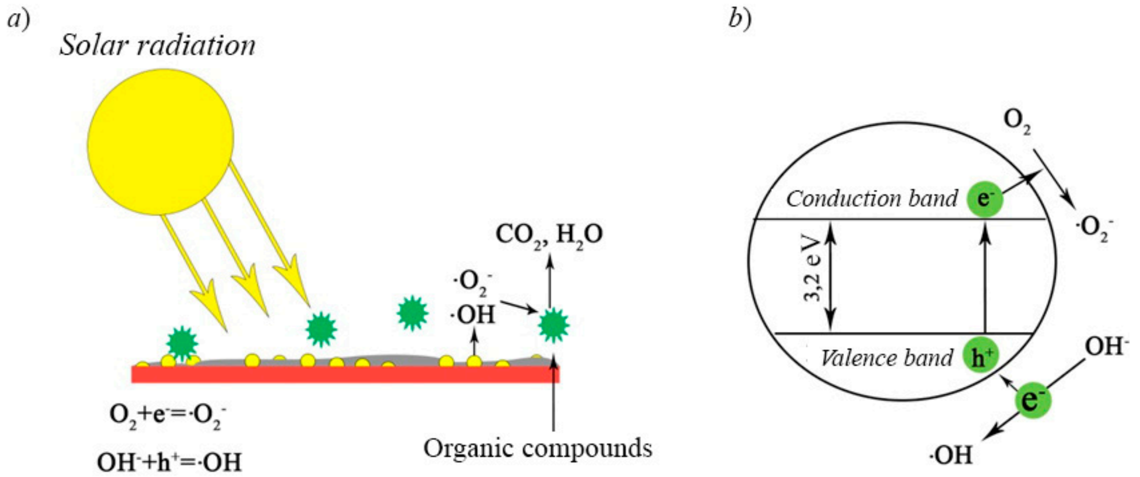

Figure S3. Mechanisms of photocatalysis (a) and energy of band gap (b)

Mechanisms of photocatalysis are shown in Figure S3. Sun can be used as an eco-friendly energy source of UV light (Figure S3, a). Primary steps of photocatalysis [5] can be classified as: (1) formation of charge carriers by a photon; (2) charge carrier recombination to liberate heat; (3) initiation of an oxidative pathway by a valence-band hole; (4) initiation of a reductive pathway by a conduction-band electron; (5) further thermal (hydrolysis or reaction with active oxygen species) and photocatalytic reactions to yield mineralization products; (6) trapping of a conduction band electron in a dangling surficial bond to yield Ti (III), and (7) trapping of a valence-band hole at a surficial titanol group. TiO_2 has the energy of band gap (3.2 eV) in the UV light, Figure S3,b.

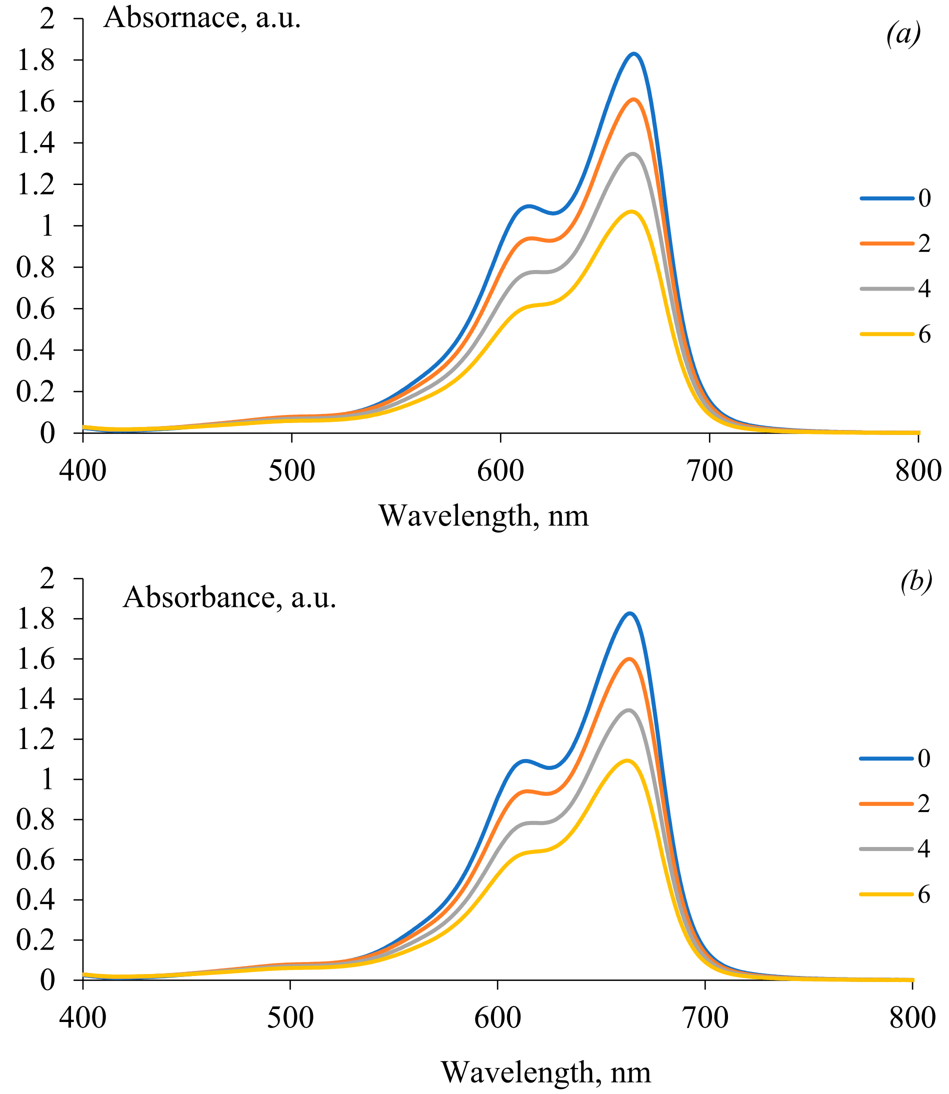

Figure S4. UV-Vis spectra of (a) Ni, (b) Ni-CeO_2 in MB aqueous solution

Table S1 Positions of XPS peaks obtained after 3.5 month corrosion tests in NaCl media

Compound	Binding energy, eV					
	Ni		Nickel−TiO_2		Nickel−CeO_2	
	before	after	before	after	before	after
Ni2p3 Scan A	852.85	853.09	852.89	852.85	853.19	853.07
Ni2p3 Scan B	857	856.5	856.5	856.5	856.7	856.7
Ni2p3 Scan C	854.53	854.9	854.62	854.9	854.35	854.7
Ni2p3 Scan D	−	−	−	861.17	−	−
O1s Scan A	531.63	531.75	531.67	531.63	531.86	531.78
O1s Scan B	530.26	530.39	530.7	530.55	530.45	530.3
O1s Scan C	533.4	533.17	533.1	533.1	533.22	533.25
O1s Scan D	528.5	528.8	529.5	528.8	−	528.65
C1s Scan A	285.04	285.03	284.91	285.21	285.18	285.06
C1s Scan B	283.76	283.96	284.23	284.11	284.23	284.08
C1s Scan C	286.88	286.63	286.58	286.88	286.62	286.73
C1s Scan D	288.46	288.46	288.46	288.46	288.5	288.61
Cl2p3 Scan A	197.21	198.7	−	197.6	−	198.37
Ni2p3 Scan D	851.26	−	−	−	−	−
Ti2p3 Scan A	−	−	458.91	−	−	−
Ce3d5 Scan A	−	−	−	−	880.96	881.42

[1] H. Zhang, Y. Li, J. Wang, N. Wu, H. Sheng, C. Chen, J. Zhao, An unprecedent hydride transfer pathway for selective photocatalytic reduction of CO_2 to formic acid on TiO_2, Appl. Catal. B Environ. 284 (2021) 119692. https://doi.org/10.1016/j.apcatb.2020.119692.

[2] K. Arifin, R.M. Yunus, L.J. Minggu, M.B. Kassim, Improvement of TiO_2 nanotubes for photoelectrochemical water splitting: Review, Int. J. Hydrogen Energy. 46 (2021) 4998–5024. https://doi.org/10.1016/j.ijhydene.2020.11.063.

[3] K. Elghniji, M. Ksibi, E. Elaloui, Sol-gel reverse micelle preparation and characterization of n-doped Tio_2: Efficient photocatalytic degradation of methylene blue in water under visible light, J. Ind. Eng. Chem. 18 (2012) 178–182. https://doi.org/10.1016/j.jiec.2011.11.011.

[4] A.J. Zimmerman, D.G. Gutierrez, V.M. Campos, D.C. Weindorf, S.K. Deb, S.U. Chacón, G. Landrot, N.G.G. Flores, M.G. Siebecker, Arsenic speciation in titanium dioxide (TiO_2) waste produced via drinking water filtration: Potential environmental implications for soils, sediments, and human health, Environ. Adv. 3 (2021) 100036. https://doi.org/10.1016/j.envadv.2021.100036.

[5] D.I. Anwar, D. Mulyadi, Synthesis of Fe-TiO_2 Composite as a photocatalyst for degradation of methylene blue, Procedia Chem. 17 (2015) 49–54. https://doi.org/10.1016/j.proche.2015.12.131.
